# Evaluation of Inactivation Methods for Rift Valley Fever Virus in Mouse Microglia

**DOI:** 10.3390/pathogens13020159

**Published:** 2024-02-10

**Authors:** Margarita V. Rangel, Feliza A. Bourguet, Carolyn I. Hall, Dina R. Weilhammer

**Affiliations:** 1 Biosciences and Biotechnology Division, Lawrence Livermore National Laboratory, Livermore, CA 94550, USA; rangel7@llnl.gov (M.V.R.); bourguet1@llnl.gov (F.A.B.); 2 Environment, Safety & Health, Biosafety & Biogovernance Functional Area, Lawrence Livermore National Laboratory, Livermore, CA 94550, USA; hall94@llnl.gov

**Keywords:** Rift Valley fever phlebovirus, inactivation, select agent, paraformaldehyde, guanidine hydrochloride, microglia, flow cytometry, arboviruses, neuropathogenesis

## Abstract

Rift Valley fever phlebovirus (RVFV) is a highly pathogenic mosquito-borne virus with bioweapon potential due to its ability to be spread by aerosol transmission. Neurological symptoms are among the worst outcomes of infection, and understanding of pathogenesis mechanisms within the brain is limited. RVFV is classified as an overlap select agent by the CDC and USDA; therefore, experiments involving fully virulent strains of virus are tightly regulated. Here, we present two methods for inactivation of live virus within samples derived from mouse microglia cells using commercially available kits for the preparation of cells for flow cytometry and RNA extraction. Using the flow cytometry protocol, we demonstrate key differences in the response of primary murine microglia to infection with fully virulent versus attenuated RVFV.

## 1. Introduction

In recent decades, arboviral epidemics with a high burden of neurological disease have occurred around the world, presenting a major biological threat to humans, livestock, and agriculture [1,2,3,4,5,6]. Rift Valley fever phlebovirus (RVFV; *Phenuiviridae* family, *Phlebovirus* genus) is a highly pathogenic mosquito-borne virus that can cause lethal disease in both humans and livestock, eliciting a wide range of clinical manifestations, including hepatitis, hemorrhagic syndrome, and encephalitis associated with various neurological symptoms [4]. Up to 17% of cases during some outbreaks display neurological symptoms, including headaches, neck stiffness, confusion, hallucinations, eye pain, and vision loss, and fatality rates among patients with neurologic manifestation of disease reach 50% [7,8,9]. Despite neurological symptoms being amongst the worst outcomes of infection, our current understanding of RVFV pathogenesis within the brain is limited. Further work is required to define mechanisms that result in clearance of RVFV from the brain versus those that contribute to damaging inflammation, the role of resident and infiltrating cells, and potential mechanisms by which the virus abrogates the host response.

Experiments aimed at gaining mechanistic insight into RVFV pathogenesis are complicated by the virus’ status as a Category A Biodefense pathogen as well as an overlap select agent by the U.S. Department of Health and Human Services (HHS) and the U.S. Department of Agriculture (USDA) [10], and the requirement to handle wild-type strains at biosafety level 3 (BSL-3) containment. Removal of infected samples from high-containment labs is often necessary to facilitate downstream analyses, which requires inactivation of live virus within those samples. The U.S. Centers for Disease Control and Prevention (CDC) established the Federal Select Agent Program (FSAP) which maintains regulatory requirements for the possession, use, and transfer of select agents, including guidance on the requirements for inactivation procedures [11]. All inactivation procedures must be validated in-house and confirmed through a pathogen viability testing protocol before implementation, and each time a select agent is inactivated for removal from registered space, inactivation of the agent must be verified based on risk assessment. The attenuated strain of RVFV MP-12, which is not a select agent and can be handled at BSL-2 containment, provides a mechanism by which pathogenesis can be studied without the need to demonstrate inactivation of virus in every sample; however, significant differences in virulence exist between strains [12], and ultimately, results obtained with MP-12 should be confirmed and/or contrasted with results obtained with non-attenuated strains.

Here, we present two methods of successful inactivation of RVFV-infected samples applied to mouse microglial cells, which are the resident immune cells of the brain. Initial tests were performed with the attenuated MP-12 strain of RVFV and methods were then validated using the wild-type ZH501 strain. First, we employed the Qiagen RNeasy Plus Mini RNA Isolation Kit for isolation of RNA from infected microglial cells. Next, we validated the inactivation of infected microglial cells using the BD Biosciences Cytofix/Cytoperm^TM^ Fixation/Permeabilization Kit for preservation of cell integrity and surface protein expression for analysis by flow cytometry. Lastly, we demonstrated the staining and analysis of surface proteins on infected primary microglia after subjecting the cells to the validated inactivation method. Results from these experiments highlight an interesting difference in the response of microglia to MP-12 and ZH501, with several surface proteins involved in immune signaling and cellular activation downregulated in cells infected with ZH501 compared to MP-12. This work helps facilitate compliance with federal regulation of select agents by providing validated methods of inactivation of RVFV-infected mammalian cells and also identifies a potential difference in immune activation between RVFV strains.

## 2. Materials and Methods

### 2.1. Cells, Mice, and Viruses

Vero, SIM A9, and LADMAC cells were obtained from the American Type Culture Collection (ATCC, Manassas, VA, USA) and maintained in culture as described previously [13]. Primary microglia were isolated from neonatal mice and cultured as described previously [13,14,15]. Microglia were maintained in culture for up to one week before use in viral infection experiments. C57BL/6J mice were obtained from Jackson Laboratory (strain #000664). All animals were housed in an Association for Assessment and Accreditation of Laboratory Animal Care (AAALAC)-accredited facility. Wild-type RVFV strain ZH501 was obtained from the NIH Biodefense and Emerging Infections Research Resources Repository, NIAID, NIH. The MP-12 strain was kindly provided by Oscar Negrete (Sandia National Laboratory, Livermore, CA, USA). RVFV stocks were propagated in Vero cells as described previously [16,17]. All work with the ZH501 strain was performed in Institutional Biosafety Committee approved BSL-3 facilities at Lawrence Livermore National Laboratory using appropriate PPE and protective measures.

### 2.2. Plaque Assay

Viral titers were evaluated by standard plaque assay as previously described [16,17]. Vero cells were seeded at a density of 250,000 cells/well in 12-well plates 1 day prior to infection. Tenfold dilutions of virus-containing samples were prepared in media (DMEM supplemented with 2% FBS, both from Thermo Fisher, Grand Island, NY, USA), and 200 μL of each dilution was used to infect cell monolayers. Cells were incubated with the virus for 30 min at 37 °C and then overlaid with medium containing 1% UltraPure agarose (Thermo Fisher, Carlsbad, CA, USA). Cells were incubated for 4 (ZH501) or 7 (MP-12) days at 37 °C and then plaques were visualized using crystal violet staining. Viral titers were determined by counting the number of plaques on the lowest countable dilution.

### 2.3. RVFV Infection of Microglial Cells

For assessment of inactivation following RNA extraction or cell fixation, SIM-A9 cells were plated in 12-well tissue culture treated plates at a density of 250,000 cells per well one day prior to infection. Cells were infected with MP-12 or ZH501 at an MOI of 8 or 10, respectively. Cells were incubated with virus for 4 h at 37 °C in 5% CO_2_. For RNA extraction, viral infection media were removed, followed by cell lysis. For cell fixation, viral infection media were removed, cells were washed one time with PBS, then replenished with fresh media and incubated for another 20 h. For assessment of microglial activation markers following RVFV infection, primary microglia were isolated from neonatal mice as described below. Cells were plated in 24-well tissue culture-treated plates at a density of 250,000 cells per well one day prior to infection. Cells were infected with RVFV MP-12 or ZH501 at an MOI of 2 and incubated for 4 h at 37 °C in 5% CO_2_. Viral infection media were removed, cells were washed one time with PBS, then replenished with fresh media and incubated for another 20 h. Supernatants were then removed, and cells were processed for flow cytometry as described below.

### 2.4. Isolation of RNA from Microglial Cells

RNA was isolated from RVFV-infected cells using the Qiagen (Germantown, MD, USA) RNeasy plus mini RNA isolation kit according to manufacturer’s instructions, paraphrased here. Cells were lysed by addition of a 350 μL buffer RLT, followed by a 1 min incubation, transfer of lysate to a microcentrifuge tube, and vortex at max speed for 1 min. Samples were centrifuged for 3 min at 8000× *g*, 22 °C. The supernatant was transferred to a gDNA eliminator spin column placed within a 2 mL collection tube and centrifuged for 30 s at 8000× *g*, 22 °C. Then, 350 μL of 70% ethanol was added to the flow-through for a total of 700 μL and mixed by pipetting. The sample was transferred to an RNeasy spin column placed within a 2 mL collection tube and centrifuged for 15 s at 8000× *g*, 22 °C. After, a 700 μL buffer RW1 was added to the column and centrifuged for 15 s as before, and then a 500 μL buffer RPE was added to the column and centrifuged for 15 s as before. An additional 500 μL buffer RPE was added to the column and centrifuged for 2 min as before. The column was transferred to a new 2 mL collection tube, and centrifuged for 1 min at 15,000× *g*, 22 °C to eliminate any residual RPE buffer. The column was placed in a new 1.5 mL collection tube; 30 μL of RNase-free water was added to spin column membrane and centrifuged for 1 min at 8000× *g*, 22 °C. The flow-through contained purified RNA in water. For validation of inactivation, purified RNA samples were split into 2 portions. One half was plated directly onto a confluent monolayer of Vero cells for evaluation via plaque assay and the other half was spiked with live virus (4E5 pfu) and evaluated by plaque assay to demonstrate that any residual chemical inactivant that may remain in the sample does not interfere with viability testing.

### 2.5. Preparation of Cells for Flow Cytometry

RVFV-infected cells were harvested by incubation in a TrypLE Express cell detachment reagent (Thermo Fisher, Grand Island, NY, USA) for 5 min. Media were added to inactivate TrypLE, and cells were transferred to a microcentrifuge tube. Cells were pelleted by centrifuging for 4 min at 500× *g* at 4 °C followed by resuspension in a 200 μL stain buffer (Hank’s balanced salt solution + 2% FBS, both from Thermo Fisher, Grand Island, NY, USA) by pipetting gently up and down. Cells were pelleted as before, then resuspended in 50 μL of a surface stain master mix (Fc block 1:100 dilution, clone 2.4G2; BD Biosciences (San Jose, CA, USA), LIVE/DEAD Fixable yellow dead stain (1:1000, Biolegend, San Diego, CA, USA)), along with the following antibodies (all from BD Biosciences, San Jose, CA, USA): CD45 APC-Cy7 (1:500, clone 30-F11), CD11b PE-CF594 (1:500, clone M1/70), CD80 FITC (1:200, clone 16-1OA1), and CD86 PE-Cy7 (1:500, clone GL1). Cells were incubated for 20 min on ice, then washed twice by addition of a 200 μL stain buffer and pelleted as before. Cells were then resuspended in a 250 μL cytofix/cytoperm solution (BD Biosciences, San Jose, CA, USA) and incubated in the dark on ice for 60 min. Cells were washed twice by addition of 750 μL of a 1× Perm/wash solution (diluted from the provided 10× solution in water) and pelleted as before. Following final wash, cells were resuspended in 200 μL 1× PBS. For validation of inactivation, cell suspension was split into 2 portions. One half was plated directly onto a confluent monolayer of Vero cells for evaluation via plaque assay and the other half was spiked with live virus and evaluated by plaque assay to demonstrate that residual chemical inactivant does not interfere with viability testing. Flow cytometry was performed using a FACSAria Fusion and data were analyzed using FlowJo software v. 10.

### 2.6. Statistical Analyses

Data were analyzed and graphed using Prism software v. 10 (GraphPad, La Jolla, CA, USA). Significance was assessed using one-way ANOVA with Tukey’s multiple comparisons test. An adjusted *p* value of 0.05 or less was considered significant, and significant differences were indicated as follows: * *p* < 0.05, ** *p* < 0.01, **** *p* < 0.0001.

## 3. Results

### 3.1. RVFV Robustly Infects Microglia

To develop an effective inactivation method for use with a wild-type strain of RVFV, we first employed the attenuated RVFV MP-12 strain, which is not a select agent and can be handled under BSL-2 containment in a non-registered space. While later experiments were performed using freshly isolated primary microglia from the brains of neonatal mice, we developed inactivation protocols using the SIM-A9 immortalized mouse microglial cell line that is easier and faster to grow than primary microglia [18]. These cells exhibit many of the same characteristics of primary microglia and have been used to study microglia behavior in the context of neuroinflammation, drug screens, and neurodegenerative therapeutic development [19,20,21,22].

In a previous study, we compared the infectivity of RVFV MP-12 in SIM-A9 cells, primary microglia isolated from neonatal mice, and Vero cells, a permissible cell line serving as a positive control. We observed a higher percentage of RVFV-positive SIM-A9 cells than of primary microglia [13]. Thus, we proceeded with validating inactivation procedures with the SIM-A9 cell line to ensure that the procedure works to inactivate the virus at a higher viral titer than we expect to obtain using primary microglia.

### 3.2. RVFV Is Inactivated during RNA Extraction from Microglia

To demonstrate that RVFV can be effectively inactivated during the extraction of RNA from samples of infected cell culture, we infected SIM-A9 cells with either MP-12 or ZH501 at an MOI of 8 or 10, respectively, and processed samples using the Qiagen RNeasy Plus Mini RNA Isolation Kit following the manufacturer’s instructions. Virus inactivation by the kit is achieved through its utilization of a highly denaturing a guanidine–isothiocyanate-containing buffer. Extracted RNA samples were split into 2 portions, where one half was plated directly onto a confluent monolayer of Vero cells for evaluation via plaque assay to demonstrate complete inactivation of the virus (denoted as Inactivated − spike), and the other half was spiked with live virus (denoted as Inactivated + spike) to demonstrate that residual chemical inactivant does not interfere with viability testing. Plaque assays revealed no observed plaques in the wells containing inactivated *−* spike samples for both MP-12 and ZH501, indicating that none of these samples contained live RVFV (Table 1), whereas inactivated + spike samples and the positive control sample demonstrated plaques that were consistent with the calculated input value. These results demonstrate that RNA extraction inactivates all RVFV MP-12 and ZH501 in our processed samples, and residual inactivant does not interfere with viability testing. Using RNA extracted from MP-12 and ZH501-infected primary microglia, we analyzed the antiviral immune response via real-time reverse transcription (RT^2^) PCR array (Qiagen, Germantown, MD, USA) and determined that there was a significant response of microglia to both viruses, although the magnitude of the response was greater to MP-12 than to ZH501 [13].

### 3.3. RVFV Is Inactivated during Cellular Fixation of Microglia

Next, we sought to validate a fixation procedure to preserve infected cells for downstream cell-based assays. Using SIM-A9 cells, we tested the BD Cytofix/Cytoperm Fixation/Permeabilization Kit. SIM-A9 cells were infected with either MP-12 or ZH501 at an MOI of 8 or 10, respectively, and incubated for 24 h. Cells were harvested and processed following the manufacturer’s instructions. The resulting samples of fixed cell suspensions were split into 2 portions, where one half was plated directly onto a confluent monolayer of Vero cells for evaluation via a plaque assay to demonstrate complete inactivation of the virus (denoted as Inactivated − spike), and the other half was spiked with live virus (denoted as Inactivated + spike) to demonstrate that residual chemical inactivant does not interfere with viability testing. Plaque assays revealed no observed plaques in the wells containing inactivated − spike samples for both MP-12 and ZH501, indicating none of these samples contained viable RVFV, whereas inactivated + spike samples exhibited plaques too numerous to count or total destruction of the monolayer (Table 2). These results demonstrate that our inactivation procedure inactivates all RVFV MP-12 and ZH501 in our processed samples, and residual inactivant does not interfere with viability testing.

### 3.4. Primary Microglia Infected with ZH501 Exhibit a Downregulation of Activation Markers Compared to Attenuated Strain MP-12

**Figure 1:**
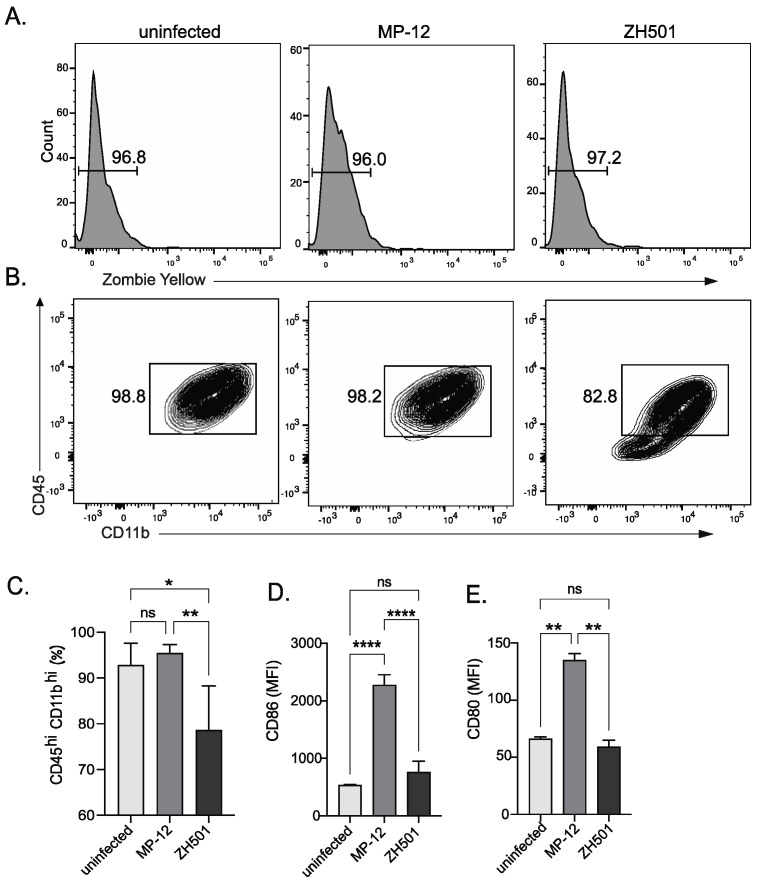
Flow cytometric analysis of RVFV-infected primary microglial cells. Primary microglia were infected with RVFV MP-12 or ZH501, and at 16 h post infection cells were harvested for flow cytometry along with uninfected controls. The percentage of live cells are indicated as zombie yellow negative (**A**). Representative plots indicated CD45 and CD11b expression (**B**). The percentages of CD45^hi^ CD11b^hi^ cells were quantitated in the indicated conditions (**C**). The expression levels of CD86 (**D**) or CD80 (**E**) were assessed on uninfected, MP-12-infected or ZH501-infected cells, as indicated. Data in C-E are shown as the mean +/− SD of triplicate samples from a representative experiment performed twice. ns = not significant, * *p* < 0.05, ** *p* < 0.01, **** *p* < 0.0001.

To demonstrate a downstream application following our inactivation procedures, we performed flow cytometric analysis to probe differences in the response of primary microglia to wild-type and attenuated strains of RVFV. Primary microglia were infected at an MOI of two with MP-12 or ZH501, or left uninfected as controls, and cells harvested and prepared for flow cytometry following a 16 h incubation. Infection with either virus did not have an impact on overall viability of the cells (Figure 1A); however, differences were noted in the expression levels of several surface markers. Expression of both CD45, a protein expressed on the surface of all immune cells, and CD11b, a protein expressed on all myeloid lineage cells, remained high on MP-12-infected cells, comparable to uninfected cells; however, a significant decrease in expression of both proteins was noted on ZH501-infected cells (Figure 1B,C). We previously reported that MP-12 infection significantly increased expression of activation markers CD86 and CD80 on the surface of microglia [13]. We aimed to verify that a similar phenomenon occurs upon ZH501 infection. Interestingly, significant increases in expression of either protein were not observed in response to ZH501 infection (Figure 1D,E), in contrast to MP-12 infection, which again resulted in high levels of CD86 and CD80 expression on the surface of primary microglia. In summary, infection of microglia with MP-12 and ZH501 yielded different outcomes, with downregulation of CD45 and CD1b and no induction of CD86 and CD80 expression in response to ZH501 infection. These results suggest a potentially important difference between RVFV genotypes that mediate the response they elicit in microglia.

## 4. Discussion

RVFV is a prominent mosquito-borne virus that poses a serious and growing public health threat. While most healthy individuals experience an acute febrile illness with minor symptoms and recover from RVFV infection, some infected individuals develop severe and often fatal neurological symptoms and meningoencephalitis. Given the severe disease outcomes involving the brain, research efforts toward better understanding RVFV neuropathogenesis and the involvement of the host immune response are crucial toward developing effective therapeutics and vaccines. RVFV is a Category A Biodefense pathogen as declared by the NIAID and an overlap select agent by the U.S. HHS and USDA. Therefore, its use for research in laboratories is regulated, and wild-type strains must be handled in BSL-3 containment facilities. Here, we provide methods to inactivate live virus in RVFV-infected samples for removal from high-containment facilities and use for downstream applications.

We first demonstrated the isolation of RNA from RVFV-infected samples using the Qiagen RNeasy Plus Mini RNA Isolation Kit and confirmed the inactivation by verifying the absence of any residual viable virus in purified RNA samples. RVFV is a negative-strand RNA virus; therefore, purified viral RNA cannot be directly translated and is not considered infectious [23]. As such, additional inactivation of purified RNA is not required to deem the samples safe for removal from registered space. We then demonstrated the inactivation of RVFV during the preparation of infected microglial cells for downstream applications such as flow cytometry using the BD Cytofix/Cytoperm^TM^ Fixation/Permeabilization Kit. While precise formulations are proprietary, the active chemicals in the Qiagen RNeasy Plus Mini RNA Isolation and BD Cytofix/Cytoperm^TM^ Fixation/Permeabilization Kits are guanidinium thiocyanate, a chaotropic agent responsible for the denaturation of proteins, and paraformaldehyde, a fixative responsible for the crosslinking of proteins, respectively. While treatments with each of these chemicals has been reported to inactivate viruses, a systematic review of their application with RVFV in mammalian cells has not been performed. We intend the documentation of these methods applied to RVFV-infected samples and the systematic demonstration of their efficacy in inactivating RVFV to aid in the selection of inactivation procedures by other groups and help facilitate compliance with the select federal agent regulations.

We employed the attenuated RVFV strain MP-12 for initial method development and wild-type strain ZH501 for validation and provided the results for both. We then used our validated cell fixation method to prepare infected primary microglia for flow cytometry. Given the fundamental functions of activated microglia during viral infection of the brain [24,25,26], we aimed to assess the expression of microglial activation markers CD86 and CD80 on microglia infected with either MP-12 or ZH501. Interestingly, MP-12 infection elicited robust expression of both CD86 and CD80, whereas ZH501 infection did not lead to significant upregulation of either protein. Previous work has demonstrated that *cd86* and *cd80* are upregulated at the transcriptional level to a greater extent during MP-12 infection of microglia versus ZH501 at 4 h post infection, which may be a downstream effect of lower-type I IFN induction by ZH501 [13]. Therefore, the lower levels of CD86 and CD80 observed may be due to an abrogation of their induction following ZH501 infection. In contrast, CD45 and CD11b are expressed on the surface of uninfected microglia [27] and their expression is downregulated upon ZH501, but not MP-12, infection. Infection with viruses from several families of DNA viruses including Herpesviruses [28,29] and Adenoviruses [30] has been shown to cause downregulation and/or modulation of CD45 activity most notably in T cells, resulting in decreased T cell activity and thus promoting viral persistence. In these cases, the modulation of CD45 activity is dependent on the specific binding of a viral protein to the CD45 protein [31]. Further work is needed to determine whether the effect of RVFV ZH501 infection on CD45 and CD11b expression is a general effect on all microglial surface proteins, or whether this is specific to CD45 and CD11b, as well as the impacts of downregulation on microglial function. Additional work is also needed to determine whether this occurs in other immune cell types and the role this plays in virulence and pathogenesis of RVFV.

In summary, this work demonstrates two methods for inactivation of RVFV within primary mammalian cells, and our results suggest that differences across RVFV genotypes dictate interactions with the host immune system that modulate outcomes such as the expression of microglial activation markers and the corresponding cell signaling and recruitment that would assist in clearance of the virus. Studies with wild-type RVFV will elucidate immune resistance mechanisms and potential viral subversion of the host immune response to approach the issue of designing new potential therapeutics more adequately.

## Figures and Tables

**Table 1:** RNA extraction inactivates RVFV. Triplicate samples of SIM A9 cells were infected with RVFV-MP12 at an MOI of 8 or ZH501 at an MOI of 10 and harvested after 4 h in culture. RNA was extracted from each sample and the split into 2, with one half plated directly onto a confluent monolayer of Vero cells for detection of live virus (Inactivated − spike) while the other half was spiked with live virus and then plated on a Vero cell monolayer (Inactivated + spike). A negative control well was treated with PBS alone. In the ZH501 experiment, a positive control well was plated with virus directly, without the addition of inactivated material. Viral growth was observed in all inactivated + spike wells with both MP-12 and ZH501. No plaques were detected in the neg control or inactivated wells. nd = none detected. TNTC = too numerous to count.

Virus	MP-12			ZH501		
Replicate #	1	2	3	1	2	3
Negative control	nd	–	–	nd	-	-
Inactivated − spike	nd	nd	nd	nd	nd	nd
Inactivated + spike	TNTC	TNTC	TNTC	34	38	24
Positive control	–	–	–	22	–	–

**Table 2:** Cytofix/cytoperm solution inactivates attenuated and wild-type RVFV. Triplicate samples of SIM A9 cells were infected with RVFV MP-12 at an MOI of 8 (for an initial protocol development trial) or RVFV ZH501 at an MOI of 10 (for 3 subsequent validation trials) and harvested after 24 h in culture. Each sample was split into 2 aliquots, with one half being plated directly onto a confluent monolayer of Vero cells for detection of live virus (Inactivated *−* spike) while the other half was spiked with live virus, then plated on a Vero cell monolayer (Inactivated + spike). A negative control well was treated with PBS alone. No plaques were detected in the neg control or inactivated wells (nd). Total destruction of the monolayer was observed in all wells containing spiked sample B. nd = none detected. TNTC = too numerous to count. TDOM = total destruction of monolayer.

Virus	MP-12			ZH501		
Replicate #	1	2	3	1	2	3
Negative control	nd	–	–	nd	-	-
Inactivated − spike	nd	nd	nd	nd	nd	nd
Inactivated + spike	TNTC	TNTC	TNTC	TDOM	TDOM	TDOM

## Data Availability

All data generated or analyzed during this study are included in this published article.
